# Promoting Wired Links in Wireless Mesh Networks: An Efficient Engineering Solution

**DOI:** 10.1371/journal.pone.0119679

**Published:** 2015-03-20

**Authors:** Behrang Barekatain, Kaamran Raahemifar, Alfonso Ariza Quintana, Alicia Triviño Cabrera

**Affiliations:** 1 Electrical & Computer Engineering Department, Ryerson University, Toronto, Ontario, Canada; 2 Dpto Tecnología Electrónica, E.T.S.I, Telecomunicación, University of Malaga, Malaga, Spain; 3 Escuela de Ingenierías, Dpto. Ingeniería Eléctrica, University of Malaga, Malaga, Spain; Nanyang Technological University, SINGAPORE

## Abstract

Wireless Mesh Networks (WMNs) cannot completely guarantee good performance of traffic sources such as video streaming. To improve the network performance, this study proposes an efficient engineering solution named Wireless-to-Ethernet-Mesh-Portal-Passageway (WEMPP) that allows effective use of wired communication in WMNs. WEMPP permits transmitting data through wired and stable paths even when the destination is in the same network as the source (Intra-traffic). Tested with four popular routing protocols (Optimized Link State Routing or OLSR as a proactive protocol, Dynamic MANET On-demand or DYMO as a reactive protocol, DYMO with spanning tree ability and HWMP), WEMPP considerably decreases the end-to-end delay, jitter, contentions and interferences on nodes, even when the network size or density varies. WEMPP is also cost-effective and increases the network throughput. Moreover, in contrast to solutions proposed by previous studies, WEMPP is easily implemented by modifying the firmware of the actual Ethernet hardware without altering the routing protocols and/or the functionality of the IP/MAC/Upper layers. In fact, there is no need for modifying the functionalities of other mesh components in order to work with WEMPPs. The results of this study show that WEMPP significantly increases the performance of all routing protocols, thus leading to better video quality on nodes.

## Introduction

A Wireless Mesh Network (WMN) consists of mesh routers and nodes that communicate by means of multiple wireless links [1]. Low installation cost, vast coverage area, self-healing and self-configuration are the most important advantages of WMNs [2]. The most significant applications of WMNs are broadband home networking, coordinated network management and community systems [1]. In some of these applications, it is necessary to access the Internet from the WMN. To allow this, mesh portals are incorporated into the wireless networks. Mesh portals come with two interfaces: wired and wireless. In the wireless medium, the mesh portals and other mesh components mainly employ the IEEE 802.11s standard [3]. This standard, which works in Layer-2 or link-layer, is an extension of the IEEE 802.11 standard for mesh networking and relies on the functionalities of the IEEE 802.11a/b/g/n technologies to carry the traffic. Multi-hop technique is included in IEEE 802.11s and allows WMNs to be more scalable than the primitive implementation of the IEEE 802.11 standard [4]. The wired interface however, is used by the conventional mesh portals only to transmit the information to a host that does not belong to the WMN. In fact mesh portals use IP (Internet Protocol) routing to access the Internet, and therefore, the outgoing traffic cannot come back to the same network. Thus, IP wired links among the mesh portals cannot be exploited for intra-traffic. This restriction imposed by IP routing can only be compromised by implementing a virtual IP connection among the gateways. This implementation is not dynamic and reconfigurations may be needed every time an IP portal is incorporated into the network.

Considering that wired links offer a higher capacity than wireless links, this paper proposes the use of wired links between mesh portals for exchanging information among nodes even when they are in the same WMN. As mentioned previously, extending the application of wired links for intra-traffic is not straightforward as the TCP/IP standards must be satisfied. This study introduces WEMPP, a Wireless-to-Ethernet-Mesh-Portal-Passageway. WEMPP incorporates special elements in the network that take advantage of available wired links. The challenge is how to include such element in the network efficiently so that it does not require modification of the other components of the network (e.g. mobile or mesh nodes). Moreover, other WEMPPs within the network should be able to dynamically auto-configure their forwarding tables to take into account the connection or disconnection of a WEMPP. Towards this goal, WEMPPs are connected in an Ethernet fashion. Additionally, a WEMPP has two main functions. Firstly, it efficiently encapsulates the received IEEE 802.11s frames into the Ethernet format without modifying or removing their sequence number. Secondly, WEMPP is able to avoid the problems imposed by the Spanning Tree Protocol (STP) [5] when two different sections (one wireless and another wired) are present in a network. Although STP or its newest version Rapid Spanning Tree Protocol (RSTP) are executed by other bridges in the wired section, WEMPPs modify some of the main procedures in these protocols to allow a frame going out of a wireless section to return to the same section through a different WEMPP. It should be noted that the mere application of an STP in a wired section closes all the ports connecting the wireless and wired medium expect one. As there is only one active port holding this condition, intra-mesh traffic cannot be rerouted through the wired links when STP or RSTP are executed without WEMPPs. Other functionalities are also incorporated into WEMPP nodes in order to improve intra-mesh traffic.

We precisely evaluate the performance of WEMPP with video traffic, one of the highly demanded traffics nowadays [6]. The network considers both mesh mobile source nodes (MSNs) and STA (Stationary) nodes. STA devices are nodes attached to access points without the capability of routing and forwarding [7]. We measured the perceived video quality on MSNs and STA nodes based on different performance metrics and in various network conditions. Specifically, we examined WEMPP's performance when the number of MSNs changes from zero to the maximum number of mesh nodes in the network. The aim was to evaluate how the most popular routing protocols in WMNs perform when WEMPPs are included in the network. Thus, we quantified the performance of Hybrid Wireless Mesh Protocol (HWMP) as a hybrid paradigm, Dynamic MANET on-demand (DYMO) as a reactive protocol [8], Optimized Link State Routing (OLSR) [9] as a proactive strategy, and DYMO with spanning tree ability as a spanning tree protocol when WEMPPs are present. Our results indicate that WEMPP improves the performance of several routing protocols in a WMN.

The rest of the paper is organized as follows. Sections 2 and 3 provide an overview of related work and the problem, respectively. The proposed solution (WEMPP) and simulation results are presented in sections 4 and 5, respectively. The paper is concluded in section 6.

## Related Work

In this section, we first analyze the limitations of the current transmission technologies. Then, we review the studies in which wired links have been employed for communication between two wireless nodes within the same WMN. Finally, we present a concise review of methods of transmitting videos in a WMN.

### A.1. Technology Overview

The purpose of our study is to connect two wireless nodes within the same network through wired links to provide a better performance than that offered by the wireless medium. To accomplish this goal, two different strategies can be adopted. The intuitive strategy would be to impose the same routing protocol in both mediums. To promote the use of wired links, the links should have a low cost in the routing metric employed by the common routing protocol. The challenge in this strategy is in selecting a routing protocol that works efficiently with wireless and wired links. Indeed, different propagation conditions have motivated the researchers to propose specific routing and transmission protocols for the multi-hop wireless networks [4,10]. Nevertheless, there are solutions such as Open Shortest Path First (OSPF) routing protocol [11] which intend to extend the original wired routing protocols to cope with wireless links. The main limitation of such approach is in employing a specific routing protocol which may not be present in an already deployed WMN or in the wired segment.

A more flexible and convenient approach is to avoid using a common routing protocol and to adapt to the existing technology. Depending on the elements with connect both mediums (they will be provided with wired and wireless interfaces), we identify two different scenarios. In one condition, the elements with connection to both mediums are routers. Routers are supported by the IP technology; the output and the input routers (also referred as gateways) will use IP to push forward the packets towards their destination. The input and output routers have to belong to the same domain to enable communication. Otherwise, the IP packets cannot return to the original domain once transferred to a different one. Although, this rule has been included in the conventional behavior of IP technology to avoid loops, it does not hold in all WMN implementations. Therefore using routers is not a feasible solution. To overcome this limitation, we could construct a VPN (Virtual Private Network) to connect the output and the input routers. Nevertheless, VPN is not dynamic in nature and would require manual configuration with every topological change in the wired section.

**Fig 1:**
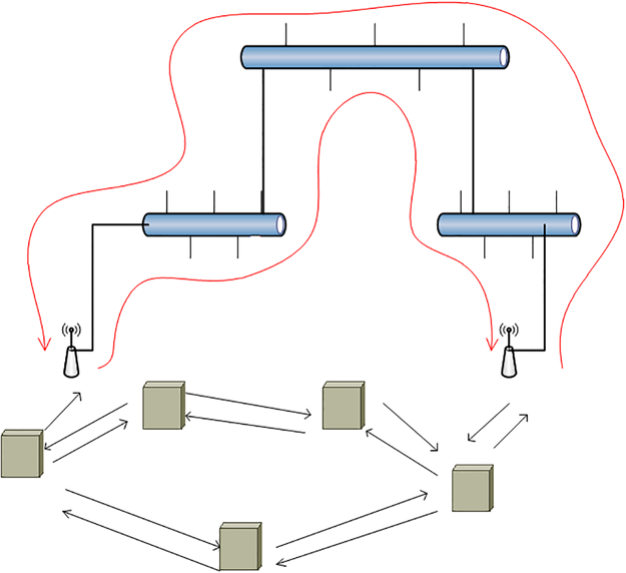
Network with Loop across the Wired Section.

Alternatively, the WMN elements connecting to the wired network could be bridges (also referred to as portals [12]). In fact, currently this is the preferred approach since many implementations for WMN are supported by Layer-2 forwarding strategies instead of routers. In this sense, IEEE 802.11s has already released its standard which is supported at Layer 2. Moreover, BATMAN [13] and Freifunk [14], which are popular WMN freeware implementations, also follow this approach. When a Layer-2 forwarding paradigm is followed, there is the possibility of creating loops. Loops cause a simple Address Resolution Protocol (APR) message to be circulated in the network indefinitely. Fig. 1 shows a loop in the wired section. To overcome this problem, Layer-2 networks commonly employ RSTP or STP. However, these protocols cannot be applied to WMN supported by wired links. We explain this limitation next.

**Fig 2:**
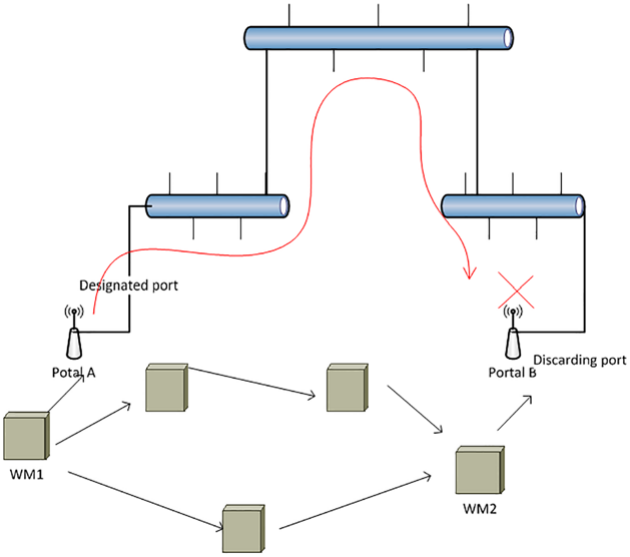
Loop Avoidance using RSTP.

RSTP avoids loops when several ports are connected to the same broadcast domain. This is done by closing all ports except the one which is referred to as the designated port in the machines. Therefore, a hierarchical tree for distribution of messages is generated. This behavior, which is desirable in wired networks, creates several problems in a heterogeneous wired-wireless Layer-2 mesh network. Specifically, RSTP only allows access to the wireless section from only one port per wired section. It is worth mentioning that in all Ethernet machines there is only one port connecting to the wired section. Fig. 2 shows the result of applying RSTP to a wired network. In particular, portal B has closed its wired port whereas portal A has configured its port as the designated one. Then, wireless frames can exclusively enter or exit the wired medium through the designated port in portal A, regardless of the number of other potentially available ports or the distance between the communicating nodes. Consequently, although WM1 may send its frames to portal A, these frames are unable to return to the wireless network. The bridge receiving a frame for the first time retransmits the frame through all its active ports except the one from which the frame has been received. Therefore, when portal B receives the frame, it is automatically discarded since its only active port is the one through which it has received the frame. Even in a more complex scenario, RTSP always ensures that there is only one active port connecting the wired and wireless sections. Thus, it is not feasible that any frame entering the wired section supported by RSTP returns to another wireless node.

As can be seen in this figure, the tree generated by RSTP clearly limits the transmission of messages. This restriction is not problematic in a purely wired section. However, in our situation it degrades the performance of the system since it prevents the two wireless nodes from relying on the wired section as a shortcut. Moreover, a node accessing the Internet is forced to employ a particular portal even when it is not the most convenient one in terms of distance, traffic, etc.

The proposed solution efficiently avoids this limitation (see Section 4). The proposed solution not only allows the nodes in the wireless network to use the wired section as a shortcut, but also guarantees the absence of loops which may cause a frame to keep circulating in the network indefinitely.

### A.2. Proposals supporting wired links for connecting wireless nodes

Ariza-Quintana et al. [15] used wired links for anycast communications. In this configuration, the portals for accessing the wired links were modified to promote the use of wired connections. Additionally, portals were dynamically selected based on an anycast communication where all portals belong to the same group. However, the authors do not provide detail information regarding the measures that need to be taken to ensure the correct behavior inside the wired section. Then, our solution is mandatory for the use of this kind of proposal. Similarly, Pande et al. propose a mathematical framework to manage mobile and wired nodes simultaneously in a Layer-2 forwarding scheme [16]. The work is focused on preserving the network topology integrity when a wireless and a wired section belong to the same domain. This is a requirement for the Authorization, Authentication and Accounting layer, which needs a common map of the neighborhood. Despite its mathematical foundations, the paper does not provide any solution about how routing is going to be deployed in such scenarios. Once again, our solution is also demanded to implement this proposal.

### A.3. Efficient Transmission of Broadcast Video Traffic

Data communication using only wireless infrastructure leads to poor network performance due to the high number of interferences and collisions common to the wireless medium [17,18]. Since wireless mesh networks are also equipped with elements connected to the Internet, some studies [19,20] have exploited the Internet backbone among the gateways for data communication between two nodes. This approach is based on the assumption that data transmission using gateways and the Internet backbone can provide better video quality on the nodes. Particularly, by employing the high capacity bandwidth on the Internet, mobile users perceive a lower end-to-end delay. However, these solutions introduce new security issues as the data is transferred through a public network such as the Internet. Moreover, the need for Internet access is an important limitation of such solutions.

Our recent studies reveal the possibility to provide high video quality using an efficient hybrid routing protocol [10] or streaming method [21]. We believe that a wired communication in WMN can also help the users enjoy smooth video playback. Such notion has been proposed in the previous studies too. For example, Fu and Agrawal [22] introduced a hierarchical architecture for utilizing random connections to Access Points (APs). The backbone links are established among Mesh Routers (MRs) and mesh portals. APs can randomly connect to MRs. Through the Internet Gateways, APs also connect to the wired Internet. Therefore, the traffic between the two nodes can be forwarded by APs using wired connections of mesh portals to which they are attached. Although this study provides an in depth mathematical and theoretical analysis of the asymptotic capacity of the proposed 3-tier hybrid WMN, its implementation is expensive and involves considerable complexity because of the following two main problems. Firstly, the implementation of Internet Engineering Task Force (IETF) [23] is not consistent with the proposed solution since it requires changing the functionality of the IP layer. Secondly, suppose that node *N*_*1*_ has two links connecting it to the wired and wireless networks. Therefore, it would be necessary to assign two different IP addresses from different IP ranges to these interfaces. Now, suppose that node *N*_*1*_ sends a packet on its wired link to node *N*_*2*_ which is in the same sub-network as *N*_*1*_ but has only a wireless interface. Such situation would require major modifications of the current IP protocol, use of VPN connection or complex manual configuration in MRs and APs in order to use three-tier hybrid WMN [22] for routing the packet from the wired interface of *N*_*1*_ to *N*_*2*_.

Aluvalu and colleagues [24] proposed another solution implemented in the link layer that allows nodes to connect with each other through wires. However, contrary to our proposed solution, in the method proposed by Aluvalu et al. a packet sent to the exterior network cannot return to an internal node in the WMN. Furthermore, none of the above studies report the results of implementation of their proposed approach. In our study however, the proposed approach has been evaluated through simulation and the results confirm that it can effectively address the aforementioned challenges.

## Problem Statement

In the previous sections, we discussed some of the existing challenges in WMNs and the recently proposed approaches to address them. The most important challenges to exploit the wired links for intra-traffic can be classified as follows:

- The effects of using the multi-hop technique on the perceived video quality on receivers in WMNs are considerable [25]. In fact the probability of packet loss, interference and collision increases as the number of hops between the source and destination increases [26]. It is worth noting that the packet loss considerably affects the performance of wireless networks [27], especially in video streaming applications. Suppose *L*_*e*_ is the packet loss probability on link *e* in a wireless network. For a path of *K* links in a multi-hop wireless communication such as the IEEE 802.11s standard, the probability of packet loss on the whole path can be calculated by Equation 1. In this equation the assumption is that there is no correlation among the losses in adjacent links. For simplicity, the probability of packet loss is considered to be the same in all links. Such simplification helps us to see that the probability of packet loss thoroughly depends on the number of hops.
  $$Packet\_Loss\_Probability_{Path}=1-\;\prod_{l=1}^{K}\left(1-L_{e}\right)$$ (1)
  As a result, it is expected that reducing the number of hops between the source and destination improve the performance of a multi-hop wireless system. A simple method to reduce the number of hops between the source and destination is to increase the transmission power of nodes, and therefore, extend their coverage area [28]. However, the problem is that *L*_*e*_ in a receiver node is correlated with the interferences provoked by other nodes. Interferences can be computed by:
  $$Interference=\sum_{N_{i}\in U}\frac{P_{i}}{A}$$ (2)
  where *P*_*i*_ is the transmission power of node *N*_*i*,_ U represents the set of nodes which can transmit in the same interval as node *N*_*i*_ receives a packet, and A represents power attenuation and is a function of the distance between the nodes. Thus, incrementing the transmission power leads to greater interference which in turn results in higher probability of packet loss. For free space propagation, Equation 2 can be written as follows [29].
  $$Interference=\sum_{N_{i}\in U}\frac{P_{i}C^{2}}{{\left(4\pi d_{i}f\right)}^{2}}$$ (3)
  where *C* is the speed of light, *f* is the frequency and *d*_*i*_ is the distance between the trans-receiving nodes. Thus, although it is necessary to decrease the number of hops and increase the transmission power to improve the system’s performance, these two operations conflict with each other. While it is possible to compute an optimal value for transmission power, the computed value will depend on various dynamic network conditions such as the number and position of nodes, network traffic, and the mobility behavior of the mesh nodes in a WMN, and therefore will not be applicable. The adjustment of the optimal transmission power requires different parameters which must be inferred from additional control messages. The inclusion of these control messages would augment the interferences and the collisions in the wireless channels. Furthermore, as the network conditions vary unpredictably, the computation needs to be repeated frequently, increasing the complexity of the solution. As an alternative solution, it is possible to divide the wireless network into *M* sub-clusters. Suppose that the network allows only nodes in the same sub-cluster to communicate with each other. In this case, packet lost probability can be calculated as follows:
  $$Packet\;Lost\;\mathrm{Pr}obability=1-{\left(1-L_{e}\right)}^{\frac{N}{M}}$$ (4)
  where *N* is the number of wireless nodes in the network.
  Although this approach considerably decreases the packet loss probability, it introduces two new important challenges into the system. The first is the challenge of connecting two nodes from different sub-clusters. The second issue is that a node can move from its current sub-cluster to another sub-cluster. Addressing these challenges is not an easy task and requires efficient algorithms and protocols. For example, the configuration of a node can be changed when it moves to a new sub-cluster. Therefore, as its first requirement, the proposed solution should be *an efficient and cost-effective solution that improves the performance of the existing routing protocols without changing their functions or the hardware of the routing devices*.
- The coverage area, network density and type of traffic are three important factors which considerably affect the amount of interference and number of collisions in a system [17,30]. For example, if the network’s coverage area increases while the density remains the same, then a packet needs to traverse more hops to reach its destination; therefore, the probability of collision rises. The same problem occurs when the number of nodes remains constant and the network density decreases. In this case, interferences and collisions in the network will sharply increase. The aforementioned can degrade the performance of the routing protocols in WMNs. Resource consumption and the effects of these existing drawbacks (e.g. Intra-Interference, Inter-Interference, collisions and contentions) increase exponentially as the number of nodes linearly grows (Equation 4). Albeit the simplest solution for this problem is to consider more sources in the system, the maintenance and management costs of such approach would be very high. Therefore, the second requirement of the proposed solution is: *It is necessary to decrease the side effects of the mentioned challenges on the performance of the existing routing protocols in WMNs*. *The solution should also be able to cope with variations in the network coverage area or the network size without increasing the amount of interferences and collisions in the system*, *that is*, *the system should be scalable*.
- Finally, mobile nodes can lead to frequent link breakages, which, in turn, trigger route repair procedures. These procedures generate control messages which may interfere with data packets. Thus *the proposed solution should be applicable even in systems with mobile mesh nodes*.

In summary:

- The solution should not elicit additional control messages and it should not require modification of the routing protocols or the hardware of the routing devices.
- The solution should offer a reasonable network performance even when the network size or density varies. Thus, it should be able to work with a cluster-based routing protocol.
- The solution should cope with the link breakages due to mobile nodes.
- Moreover, the solution should be feasible, cost-effective and technically implementable in the current WMNs. For example, modification of the IP routing protocol is not considered a valid solution.

In order to fulfill these requirements, we propose using WEMPP, explained in detail in the next section. Moreover, the simulation results show how incorporating WEMPPs improved the performance of different routing protocols.

### Wireless-to-Ethernet Mesh Portal Passageway (WEMPP)

**Fig 3:**
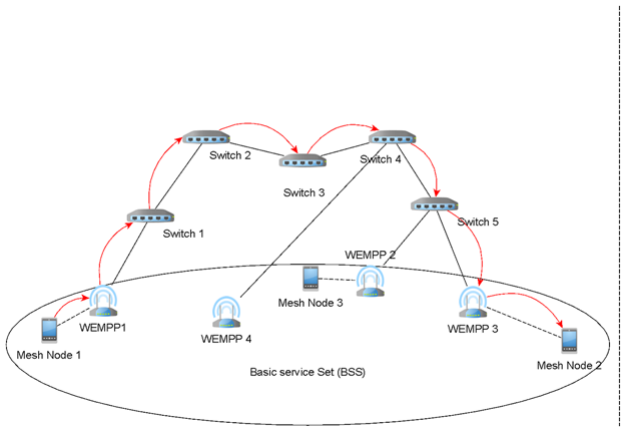
Mesh node 1 is connected to mesh node 2 using WEMPPs (Physical View).

**Fig 4:**
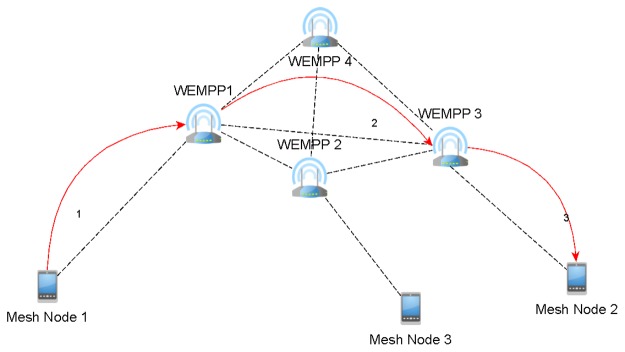
Mesh node 1 is connected to mesh node 2 using WEMPPs (Logical View).

**Fig 5:**
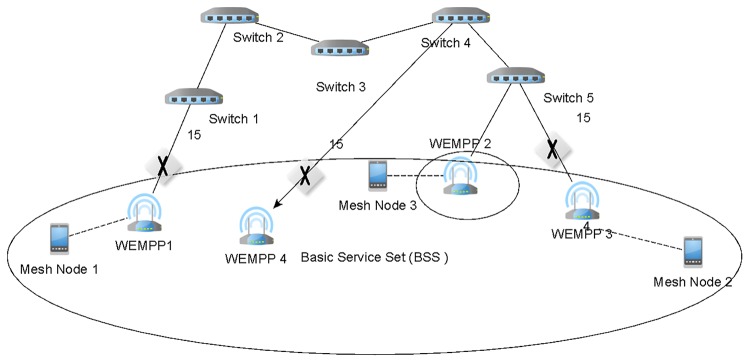
RSTP discarding Ports on WEMPPs.

Wireless to Ethernet Mesh Portal Passageway (WEMPP) is based on the Ethernet technology. A WEMPP is a mesh portal device capable of connecting to other mesh portals in a wireless mesh network or to another WEMPP using wired communication. It has at least two interfaces: a wired one and a wireless one. The wired communication is an Ethernet IEEE 802.3 technology. In order to understand how WEMPP works, we differentiate its operation under the following circumstances:

- **WEMPP forwarding unicast data**: When WEMPP receives a unicast frame from a mesh node, depending on the next hop towards the destination it performs one of the following two tasks. If the next hop found on its routing table is a WEMPP too then it will directly encapsulate the received IEEE 802.11s frame over the Ethernet frame format. Otherwise, the WEMPP will act as a conventional mesh node and sends the frame to a mesh node without encapsulation over the Ethernet format. In fact, a mesh node can find the nearest WEMPP using its routing table. Considering Figs. 3 and 4, suppose that mesh node 1 intends to send a frame to mesh node 2. Without WEMPP, mesh node 1 should send the frame using unreliable wireless infrastructure in the WMN. Even if there were some conventional mesh portals connected to the Internet, the communication should be supported by the wireless infrastructure as IP routing restricts receiving a packet that was generated in the same network. Using the WEMPP framework, the source sends the frame to WEMPP1. WEMPP1 immediately finds the nearest WEMPP to mesh node 2 (WEMPP3) using a routing protocol.
- **WEMPP forwarding broadcast messages**: When the WEMPP receives a broadcast message, it duplicates the broadcast packet towards each WEMPP as the next hop using unicast communications. As explained below, in the auto-configuration process, each WEMPP knows about other WEMPPs which is explained next.
- **WEMPP data encapsulation:** When WEMPP uses wired links, it encapsulates the received frame into the Ethernet format. In particular, the device indicates in the ‘Ethertype’ field of the Ethernet frame that it is a 802.11s. Encapsulating has significant benefits over frame conversion. Specifically, encapsulation does not require removing the frame sequence number. Recall that, direct conversion of IEEE 802.11s to Ethernet format removes the sequence number of the frame leading to multiple retransmissions of the frame before it arrives at the destination. This operation would cause a high network overload. However, WEMPP efficiently avoids this problem by encapsulating the IEEE 802.11s frame in the IEEE 802.3 (Ethernet) technology. By inserting the IEEE 802.11s frame in an IEEE 802.3 frame all the IEEE 802.11s fields, including the sequence number, are preserved. For our example, WEMPP1 sends the data to the Ethernet address of WEMPP3 through the wired segment. As soon as WEMPP3 receives the frame, it converts the Ethernet to IEEE 802.11s format and sends it to mesh node 2.
- **WEMPP data fragmentation**: Taking into account that IEEE 802.11s allows a Maximum Transfer Unit (MTU) of 2300 bytes whereas IEEE 802.3 allows only 1500 bytes, WEMPPs are designed to fragment an IEEE 802.11s frame into several IEEE 802.3 frames if necessary.
- **WEMPP auto-configuration:** When a new WEMPP is incorporated into the network, it must discover other WEMPPs. To do so, the new WEMPP broadcasts an IEEE 802.3 message that will be received by other WEMPPs through the wired or wireless links. Particularly, we have implemented the message by setting the Ethertype field to WEMPP. WEMPP is encoded as 0x820 in the Ethertype field, which is currently unused. Alternatively, the receivers announce their availability by another IEEE 802.3 broadcast message whose Ethertype field is also set to WEMPP. Then, the newly joined WEMPP must keep an up-to-date list of active WEMPPs in the network. This is done through passive and active methods. In the passive approach, the WEMPP employs any traffic coming from the neighboring WEMPPs to update the list. Since the IEEE 802.11s routing protocols periodically generate messages which are forwarded into the Ethernet by WEMPPs, this is the most common way to keep an updated list of neighboring WEMPPs. The WEMPPs may proactively broadcast an availability message, as a new WEMPP does when it joins the network.
- **WEMPP adaptation to STP or RSTP**: As explained in Section 2, it is necessary to include a protocol that avoids loops when there are multiple switches in the Ethernet. Therefore, STP or RSTP are mainly incorporated into the IEEE 802.3 technology (Fig. 5).
  These protocols just let an active port among all the nodes connecting the wireless and the wired domains. As shown in Section 2, the mere application of STP or RSTP in the WEMPP framework would not allow proper communication among WEMPPs; rather it requires some modifications. However, the modifications are required only in the WEMPP nodes. The STP/RSTP execution in other Ethernet nodes does not require any modifications.
  The first modification is to ensure the active port connecting the wireless and wired domains belongs to a WEMPP. Here we take advantage of the option provided by RSTP for assigning priorities to the network nodes. A higher priority implies that the node will be placed in upper levels in the tree generated by the RSTP. Specifically, one WEMPP is said to have the highest bandwidth, which is equivalent to highest priority. The selection of the WEMPP with the highest RSTP priority could attend to diverse criteria such as the distance to other nodes, economical reasons or performance issues.
  The remainder WEMPP nodes have all their ports connecting to the wireless medium blocked. A conventional bridge would discard all the frames generated in the wireless domain following the RSTP rules in these port-blocked machines. However, a WEMPP does process particular Ethernet frames. Specifically, the considered frames should fulfill any of the following conditions:
  - The destination address of the Ethernet frame should be the same as the WEMPP node that is processing the frame.
  - The frame should encapsulate an IEEE 802.11 frame. This can be easily confirmed by checking the Ethertype field. If the aforementioned two conditions are met, the WEMPP node can retransmit the frame to the wireless medium through an even disabled port. In order to avoid loops, the WEMPPs check the sequence number associated to the IEEE 802.11 frames. Note that our engineering solution preserves the sequence number of the IEEE 802.11 frames by encapsulation. In fact, encapsulation is necessary to guarantee successful route discovery in the IEEE 802.11 protocol.
  - The Ethernet frame is a message used for the WEMPP discovery process.

By setting the previous procedures in the WEMPP nodes, our solution promotes using a wired segment even for connecting two wireless nodes in the same network. The wired segment is more reliable and provides a higher bandwidth than wireless links. Figs. 3 and 4 depict this infrastructure from physical and logical viewpoints, respectively. Here, mesh node 1 sends a frame to mesh node 2 without knowing any wireless route to mesh node 2. However, mesh node 1 knows that mesh node 2 is attached to a WEMPP. In fact the frame will be sent from WEMPP1 to WEMPP3 (indicated by the red arrows) using a routing protocol such as AODV. One of the biggest advantages of using WEMPP is that it allows a WMN to reduce the number of hops between the source and destination while the interferences supported by the wireless medium can be decreased. As mentioned earlier, the number of interferences, contentions and collisions increases as the number of hops between two nodes increases. Thus, the WEMPP framework reduces the number of lost packets and, in turn, allows a better video quality on mesh nodes, for instance. In addition, the wireless mesh network is more scalable when WEMPPs are used. With WEMPP communications are supported by shorter paths, therefore, it is possible to increase the network density without significantly increasing the interferences in the system.

WEMPPs should only be used when it is more convenient to use wired links for intra-traffic between two WMNs. The convenience is determined by the routing metric so no all intra-traffic necessarily traverses the Ethernet. WEMPPs are only other WMNs in the network but they are connected to other WEMPPs by means of our proposed solution.

Another advantage of the proposed solution is that it can be implemented without any changes in the upper OSI (Open System Interconnection) layer protocols. Furthermore, WEMPP allows nodes in different sub-clusters to communicate with each other and move from one sub-cluster to another without requiring any reconfiguration in the node. The proposed solution does not modify the functions of the IP layer. In fact, the only requirement for using WEMPP is to change the firmware of a switch. WEMPP can be easily implemented by modifying the firmware of the actual Ethernet hardware.

In summary, WEMPP is a cost-effective engineering solution because:

- It significantly improves the performance of the system by using wired links to transmit intra-mesh traffic.
- It is conformant with the current protocols and does not require any changes in the actual deployed network.
- It requires no modification of the actual devices. It only requires a small modification in the actual firmware of the WEMPP. This modification is in the behavior of the portals which are about to be incorporated, not in the already deployed protocols or devices.

To understand the effects of employing WEMPP in a WMN on the performance of the existing routing protocols, a simulation was carried out based on the INETMANET framework in OMNET++ [31]. WEMPP was carefully implemented in the simulator to show how it can improve the performance of the existing routing protocols without changing their algorithms or functions. Changes in a routing protocol are costly and they often bring along new challenges.

## Simulation

In this section, the performance of a system including WEMPPs is measured carefully and precisely using OMNET++, a discrete event-based simulator. OMNET++ is a modular, extensible and component-based simulator consisting of several C++ simulation libraries and frameworks. It was originally designed for building network simulators including wired and wireless communication systems, queuing networks, on-chip networks and so on. Moreover, the existing frameworks in OMNET++ provide flexible and reliable features for simulating wireless sensor networks, wireless mesh networks, various Internet protocols, photonic networks and performance modeling. The INETMANET framework is based on the INET framework and is designed for modeling different wireless communications and systems in OMNET++. The simulation parameters and results are presented and discussed in the following sub-sections. Specifically, we evaluate how different routing protocols perform when WEMPPs are incorporated into the wireless mesh networks.

### B.1. Simulation Parameters

**Fig 6:**
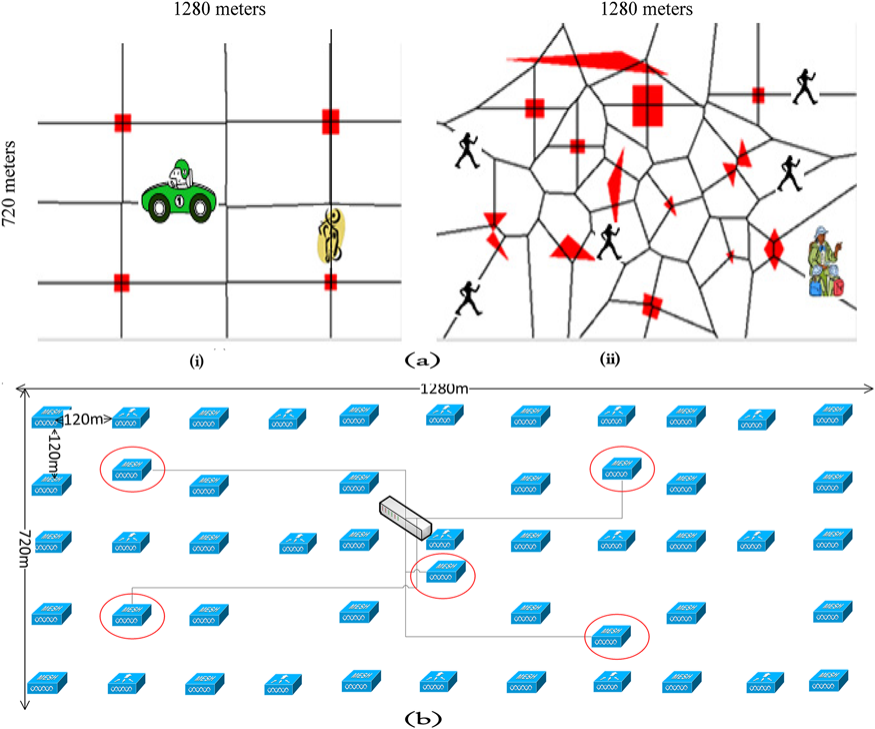
Node Mobility Model in the Designed Park Environment for Mesh (i) and STA (ii) Nodes and Designed Backbone for Hybrid WMN (WEMPP Nodes are Marked by Ellipse) (b).

The simulation parameters and their values are summarized in Table 1. We have chosen video traffic since it has the highest demand in today’s world [7,32]. A video server, placed as a node, distributes 2000 seconds of live video stream [33] to the network. To provide a precise mobility model for STA and mesh nodes, we have designed and implemented RWWP (Random WayPoint Within Pre-defined path) model using PathAdHoc [34]. In contrast to the RWP (Random WayPoint), which has been used in many of the previous studies such as [35], PathAdHoc allows RWWP to provide a more realistic mobility model for WMNs. In fact, OMNET++ uses the generated output file of this tool as the input parameters for simulating the designed park in Fig. 6a. The figure shows a 1280×720 square meter park including some obstacles which were precisely designed using PathAdHoc tool. The red areas represent the obstacles, and the lines represent the pathways. Fig. 6b depicts the backbone used in this simulation. WEMPPs, marked by ellipse, are connected to each other using solid lines (wired links).

**Table 1:** Considered Parameters in the Simulation.

Parameter	Value(s)
Simulation Time	2000 Seconds
Packet Size	512 Bytes
Video Stream	Silence of the Lamb available from
Network Area Size	1280×720 m^2^
Infrastructure	Hybrid Wireless Mesh Network
Mac Layer Standard	IEEE 802.11g
Distribution Model	Two Ray
Interference Model	Additive
Number of STA Nodes	10
Number of Mesh Nodes	10
Node Mobility Model	RWWP (Random Waypoint Within pre-defined Path)
Mesh Mobility Speed	Uniform(5,15) mps
Pause Time for Mesh Nodes	Zero second
STA Mobility Speed	Uniform(0.5,1.5) mps
Pause Time for STA Nodes	uniform(0,600) second(s)
Rate of MSNs	10, 20, 40, 70, 90, 100 Percent of Mesh Nodes
Routing protocols	HWMP, Proactive (OLSR), Reactive (DYMO), Spanning Tree (DYMO)

When a node reaches a border, it stops, turns and continues in the opposite direction. We assume that mesh nodes are in high speed vehicles such as cars and motorbikes with their movements restricted by the road (Fig. 6a.i). Moreover, the assumption is that all pedestrians (i.e., STA nodes) use their wireless devices such as Smartphones and Tablets and stay on the paths determined by Voronoi analysis [36] of the area with obstacles (Fig. 6a.ii). All STA nodes, i.e., active clients, receive the distributed video stream from the video server. In addition, some MSNs request video traffic from the video server. MSNs are randomly selected from the pool of existing mesh nodes. As shown in Table 1, the number of MSNs changes based on the number of existing mesh nodes in the network.

The goal of simulation was to examine how some well-known routing protocols intended for wireless mesh networks cope with WEMPPs. Particularly, we evaluated: (i) OLSR as a proactive routing protocol, (ii) DYMO as a reactive protocol, (iii) DYMO configured as a Spanning Tree, and (iv) HWMP as one of the routing protocols specified in IEEE 802.11s. We disabled the path accumulation feature in DYMO in its reactive mode. Therefore, its behavior is similar to that of AODV which is a reactive routing protocol. Moreover, DYMO has been employed as the basis to model a spanning tree routing protocol. In this case, the root sends a Route Request packet which will be broadcasted by the intermediate nodes and will be immediately answered with a Route Reply. Table 2 provides detailed information about the routing protocols used for simulation. These routing protocols run in the link layer.

**Table 2:** The characteristics of the considered routing protocols in the simulation.

Type	Protocol	Source Code	Characteristics
Proactive	OLSR	NS3, OLSR Implementation	Hello Interval = 2s, TC Interval = 5s
Reactive	DYMO	DYMO-UM	Accumulated path is disabled
			Gratuitous Route Reply is disabled
			Link-Layer feedback is active for detecting path failure
Spanning Tree	DYMO	DYMO-UM	Spanning tree proactive mechanism
			Minimum number of hops
			Proactive Timeout = 5s
HWMP	HWMP	NS3, HWMP Implementation	Based on the IEEE 802.11–2012 specification
			Root node is enabled and embedded in the video server
			Only in reactive mode, the min hop cost is used instead of the RA metric
			Link-layer feedback is active to detect path failure

### B.2. Simulation Results and Discussion

This study evaluates the efficiencies of the routing protocols listed in Table 1 with and without WEMPP when the number of MSNs varies. This is done by examining the following five variables:

- Total successfully received video Packets (TSRP): TSRP refers to the total number of successfully received video packets in a node. These are accurate packets received before their playback time,
- End-to-End Delay (EED): the delay in transferring a packet from the source to destination,
- Packet delay variation (PDV): PDV or jitter is the difference in EED one-way delay between the selected packets in a video flow with any lost packet being ignored. PDV was calculated for each packet and the average PDV of all packets in STA or MSN nodes was calculated for different routing protocols,
- Routing overhead: the ratio of the number of useless packets to the total number of transmitted packets by a routing protocol,
- Video distortion: the capacity of non-successfully played video frames in byte divided by the total capacity of all video frames expressed in percentage.

**Fig 7:**
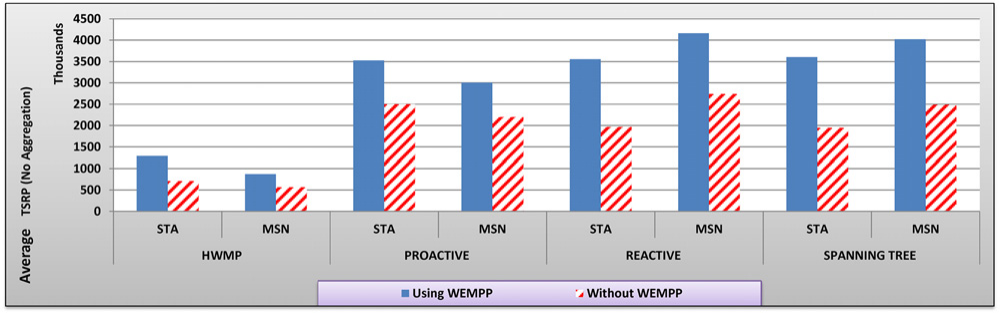
The average TSRP in different routing protocols with and without WEMPP.

**Fig 8:**
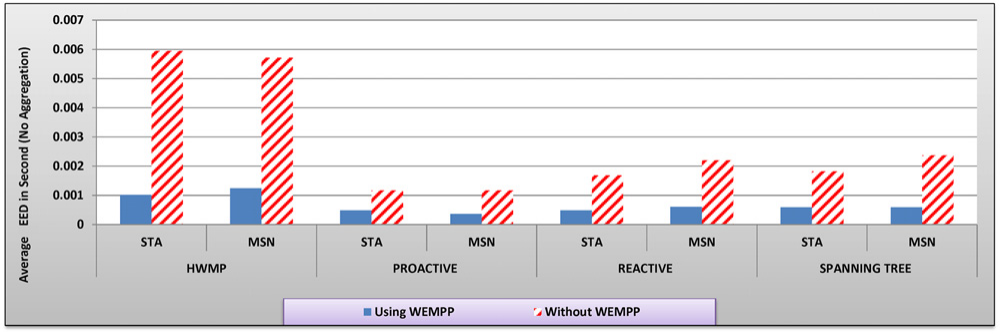
The average EED (s) in different routing protocols with and without WEMPP.

**Fig 9:**
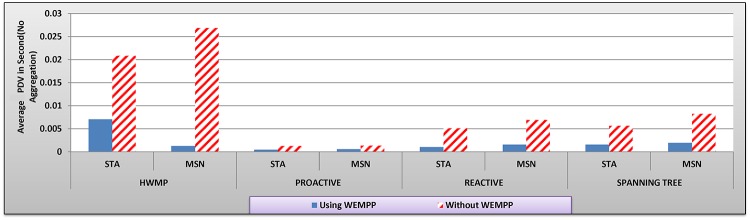
The average PDV (s) in different routing protocols with and without WEMPP.

**Fig 10:**
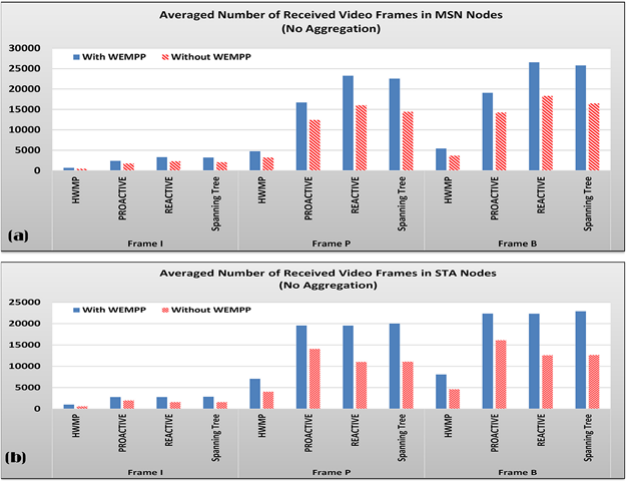
Average amounts of Received Frames in Mesh nodes With and Without WEMPP (a) and in STA nodes With and Without WEMPP (b).

**Fig 11:**
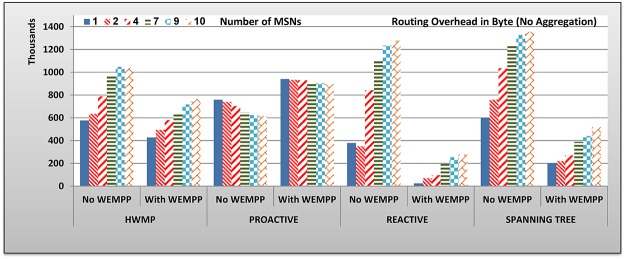
Imposed Routing Overhead by Routing Protocols on MSNs.

**Fig 12:**
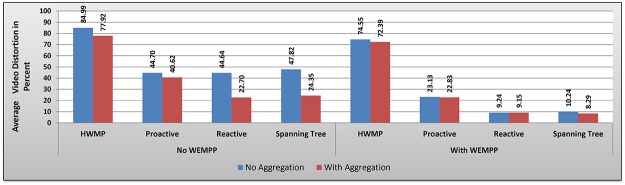
Video Distortion Percentage with and without WEMPP.

The video stream has a G16B1 structure in which each Group-of-Pictures includes 16 frames. The frame frequency is 30 per second and the rate of MSN changes from 10 to 100 percent of mesh nodes in increments of 10. To examine the efficiency of WEMPP in live video streaming, we ran the simulation 5 times and calculated the average values of each variable of interest (Figs. 7 to 12). In these figures, no aggregation means that each packet includes only one or a part of one video frame while aggregation mode means each packet can contain more than one video frame.

Fig. 7 shows that STA and Mesh nodes receive a much higher number of video packets when WEMPP is used; a clear indication that WEMPP can be useful for live video streaming. When WEMPP is used in the network, STA nodes show 81.70, 41.02, 80.86, and 85.05 percent improvement in TSRP for HWMP, proactive, reactive and spanning tree routing protocols, respectively. Similarly, MSN showed 54.25, 36.69, 51.85, and 61.82 percent improvement in TSRP for HWMP, proactive, reactive and spanning tree routing protocols, respectively. These results clearly show how WEMPP increases video quality on nodes, because they can buffer more number of video packets before their playback times. HWMP routing protocol cannot provide good performance when there are mobile nodes in the network [7]. However, all nodes improve the most by using WEMPP when HWMP is employed in the network.

Adding WEMPP to HWMP protocol considerably reduces the EED to less than 1 ms (Fig. 8). When WEMPP is used in the network, STA nodes show 82.82, 58.22, 70.97, and 67.50 percent improvement in EED for HWMP, proactive, reactive and spanning tree routing protocols, respectively. As the result, these nodes can receive the required video packets in proper time which leads to less video distortion. MSN also showed 78.25, 68.26, 72.47, and 75.09 percent improvement in EED for HWMP, proactive, reactive and spanning tree routing protocols, respectively. Node mobility can noticeably affect the performance of proactive protocols. However, WEMPP addresses this problem by efficiently routing the video packets among nodes using a wired segment which is not degraded by mobility (Fig. 8).

Fig. 9 clearly shows that WEMPP also significantly reduces PDV in a WMN when a live video stream is distributed. HWMP benefits from WEMPPs to a greater extent, showing 66.11 and 95.38 percent improvement in PDV for STA and mesh nodes, respectively. When WEMPP is used, STA and mesh nodes also show on average 60.05, 76.99, and 74.3 percent improvement for proactive, reactive and spanning tree routing protocols, respectively.

In summary, Figs. 7 to 9 show that WEMPP is slightly more beneficial for STA nodes. Since STA nodes constitute a large fraction of nodes in real WMNs, WEMPP are considered an important component of these networks.

Although Figs. 7 to 9 confirm that WEMPP increases the quality of a live video stream, it is necessary to measure its efficiency in terms of the total number of received video frames. Fig. 10(a) and 10(b) show the average number of received video frames (I, P and B) in Mesh and STA nodes with and without WEMPP, respectively. Both STA and Mesh nodes receive more video frames when WEMPP is used in the network. In other words, STA and mesh nodes averagely experience 68.96 and 46.66 percent improvement in this metric, respectively. When a node receives more video frames, the probability of playback skip due to frame dependency sharply decreases [37].

Fig. 11 shows that WEMPP decreases the magnitude of the imposed routing overhead on the system by different routing protocols. Routing overhead is improved by 28.39, 82.28, and 67.71 percent in HWMP, reactive and spanning tree routing protocols, respectively. On the other hand, routing overhead is increased by 34.69 percent when WEMPP is used in the network. Proactive imposes higher routing overhead, because there are more nodes that are neighbors when WEMPP is used. In fact, WEMPP nodes exchange routing messages among these nodes leading to more routing overhead. In fact a greater number of neighboring nodes translates to larger OLSR packets. The Hello Message includes the list of neighboring nodes and the WEMPP nodes are included in the list of neighbors of all the WEMPP nodes. Without WEMPP these nodes are not neighbors and, therefore, are not included in the neighbors list. So smaller messages in size are exchanged.

In live video streaming, WEMPP considerably affects the performance of HWMP for MSNs (Figs. 7 to 9). Recall that, HWMP is designed for stationary nodes and cannot provide high performance in dynamic WMNs where nodes constantly move.

Video distortion is an important indication of the quality of live video streaming. Based on Fig. 12, WEMPP improves the quality of video by reducing distortion. Given its importance, we measured video distortion in two cases. In the first case, aggregation was not used in the network, i.e., each packet could include only one or a part of one video frame. Using WEMPP, all routing protocols introduced less video distortion to the network. In other words when aggregation method was not used in the network, WEMPP reduced video distortion by 12.28, 48.25, 79.31, and 78.58 percent for HWMP, reactive, proactive and spanning tree routing protocols, respectively. Using aggregation method, WEMPP reduced video distortion by 7.10, 43.80, 59.69, and 65.97 percent for HWMP, reactive, proactive and spanning tree routing protocols, respectively. The aforementioned results mean that on average video distortion was reduced by 54.61 and 44.14 percent without and with aggregation method, respectively. The fact is more packets can be generated and exchanged in the network when aggregation method is not used. With more packets in the network, the traffic and in turn the number of collisions will increase too. Altogether, the results clearly show that WEMPP can efficiently address these problems. Considering the aforementioned results we believe WEMPP is an efficient and feasible solution for video streaming over WMNs.

## Conclusion

In this study, we introduced WEMPP as an efficient engineering solution to resolve some of the most significant problems in WMNs, namely reduced bandwidth, poor communication and low scalability. WEMPPs are special elements with wireless and wired interfaces that promote the use of wired links even for intra-traffic where the source and destination of the traffic within the same network. Particularly, WEMPPs are connected through an Ethernet, which is characterized by a higher bandwidth than the wireless medium. WEMPPs are carefully designed so that they can be employed by other elements of the network without the need for any change in their functionality. Moreover, the WEMPP framework is applicable to diverse routing protocols. We showed that regardless of the used routing protocol and the type of distributed video stream, WEMPP improves the network performance by reducing the end-to-end delay, the packet delay variation, and the routing overhead. The aforementioned changes increase the total number of successfully received video packets (TSRP) and, consequently, reduce video distortion. Moreover, WEMPP attenuates the detrimental effect of having multiple mesh source nodes on the perceived video quality. Our results clearly show that employing WEMPP keeps the system in a more stable condition, and improves the dynamic behavior of the network due to the higher mobility rates of the nodes. As a result, the perceived video quality on both STA and mesh nodes considerably improves. Furthermore, there is no need to change the functions of IP layer and routing protocols. In fact, WEMPP is an engineering solution that effectively improves the performance of the existing routing protocols without imposing additional costs.

In our future work, we plan to implement a test-based experiment. We will measure the dynamic performance of the proposed solution (e.g. the required time for detecting a new WEMPP or any change in the wired section). Moreover, we will measure how our solution adapts to changes such as incorporating a WEMPP or turning off the WEMPP.
